# Remodulin® Pump Failure: An Emergency Medicine Simulation Scenario

**DOI:** 10.7759/cureus.8223

**Published:** 2020-05-21

**Authors:** Steven Wipprecht, Jake Wagner, Anna Bona, Lauren Falvo, Rami A Ahmed

**Affiliations:** 1 Emergency Department, Indiana University School of Medicine, Indianapolis, USA; 2 Emergency Medicine, Indiana University School of Medicine, Indianapolis, USA; 3 Emergency Medicine, Methodist Hospital, Indianapolis, USA

**Keywords:** remodulin, pulmonary hypertension, simulation, critical care, pulmonary critical care, simulation in medical education, emergency medicine, treprostinil

## Abstract

Pulmonary hypertension (PH) is a progressive disease that causes high patient mortality. With limited hemodynamic reserve, many PH patients require maintenance IV infusion medications to maintain their activities of daily living. One common delivery method for this targeted therapy is through a Remodulin® (treprostinil) pump. When presenting for emergent evaluation, decompensating PH patients have a broad differential diagnosis including pump failure. PH patients are at a high risk of poor patient outcomes given the difficulty in recognizing PH-specific symptoms and unique aspects of their management. Therefore, learners will benefit from participating in an immersive simulation-based PH patient scenario in a safe learning environment. Here, we present a simulated scenario of a decompensating PH patient on a Remodulin® pump.

## Introduction

Pulmonary hypertension (PH) is a progressive disease that causes high patient mortality. It is defined as a mean pulmonary artery pressure of ≥25 mm Hg at rest [1]. PH includes a heterogeneous group of disorders that are differentiated based on clinical, hemodynamic, and histopathologic features including embolic, respiratory, venous, and arterial disease [2]. The estimated prevalence of PH is about 1% of the global population, which increases up to 10% in individuals above the age of 65 [3]. Patients suffer from high mortality secondary to severely blunted hemodynamics and little pulmonary reserve, with an overall 58% survival rate at three years [4]. In addition, patients experience difficulty in performing daily tasks due to shortness of breath, swelling of the legs and abdomen, and progressive fatigue. While recent advances in care have improved PH patients' quality of life, few measures are truly curative.

One subset of PH, pulmonary arterial hypertension (PAH) occurs when elevated pressures are caused by an obstructive environment in the small arteries of the lung. The cause is often unknown but can be due to medications, HIV infection, connective tissue/autoimmune disorders, and other related factors [5]. Targeted therapies have shown benefits in impacting PAH patients' functional status and daily living conditions. Remodulin®, an IV infusion of treprostinil, is one such therapy used in the treatment of PAH. It acts through the direct vasodilation of pulmonary arterial vascular beds. This reduces pulmonary arterial pressures, therefore increasing cardiac output (CO) and systemic oxygen distribution. These effects are achieved while having minimal effect on heart rate [6,7]. Simonneau et al. found that Remodulin® improved symptom relief and hemodynamics compared to placebo with a composite week 12 post-treatment baseline of 8.5±0.5 compared to 7.4±0.2 in placebo, p<0.0001 [8]. Side-effects from medications like Remodulin® often stem from the mode in which they are delivered and their mechanism. These can range from a mild sensation of feeling flushed to severe infusion site discomfort. Given its roughly four hour half-life, abrupt cessation of Remodulin® can cause noticeable hemodynamic compromise in as early as two hours [9]. Up to 10% of patients in the initial 2017 Remodulin® study had some form of pump failure after four years and 33% had no corresponding alarm to alert them [10]. Abrupt pump failure leads to PH patients presenting in extremis with progressive symptoms of dyspnea, edema, angina and signs such as tachycardia, hypoxia, and hypotension [10,11].

The profound variability among PH patients' baseline vital signs and clinical exams makes it difficult for providers to recognize acute changes. Even more challenging for the treating provider is the nuanced nature of the targeted therapies. Suboptimal treatment due to the misunderstood and unique pathology of PAH can worsen the patient’s clinical condition and must be avoided at all costs. Kingman et al. interviewed over 100 PH nurses and clinicians and found that 68% of respondents reported serious or potentially serious errors in PAH medication administration [12]. This study highlights the need for standardization of hospital treatment protocols and the need for further education in clinical training environments [12]. Optimal treatment typically includes limiting fluid resuscitation to 250 mL and is only indicated if the patient is dehydrated. Maximization of oxygenation is imperative. Use of non-invasive oxygenation (non-rebreather, high-flow nasal cannula) should be the initial resuscitation strategy to limit hypoxia. Vasopressors should be utilized early to maintain adequate oxygen delivery. If these modes fail, only then should more invasive modes of positive pressure ventilation be used with the lowest possible pressures [13]. Early communication and inclusion of PH coordinators and specialists is essential. The purpose of this simulation is to provide a guide for learners to recognize the signs and symptoms of a crashing PH patient, familiarize themselves with the common medications and various available pumps, understand the intricacies of a patient presenting with Remodulin® pump failure, and acquire the appropriate management skills needed to improve patient outcomes and prevent patient harm.

## Technical report

This simulation scenario is designed to occur in a rural emergency setting. Department capabilities include ability for advanced cardiac life support with standard airway resources, defibrillator and code cart, continuous ECG and vital sign monitoring, and a high-fidelity simulator. Learners have access to all standard code cart medications as well as laboratory studies and radiography. Given the rural environment, hospital specialists are only available via consultant calls to surrounding outside hospitals.

**Figure 1:**
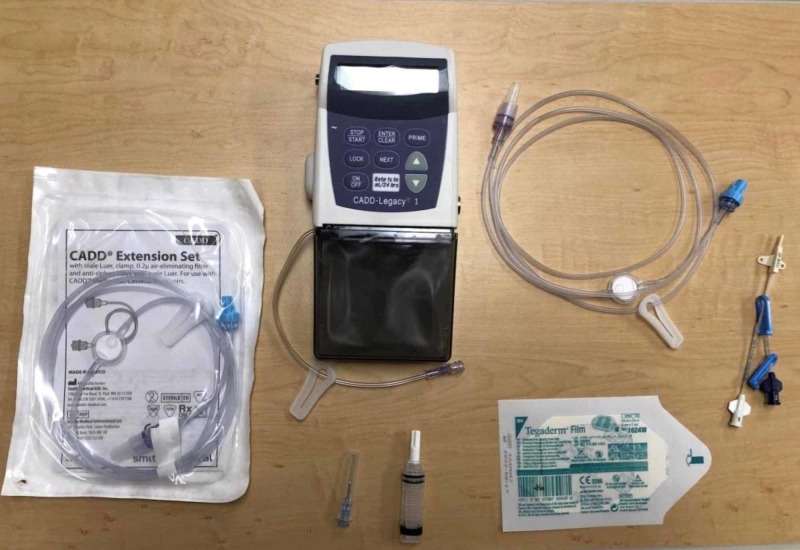
Intravenous Remodulin® pump supplies CADD-Legacy® 1 pump with IV tubing, central line supplies, and Tegaderm™ film

**Figure 2:**
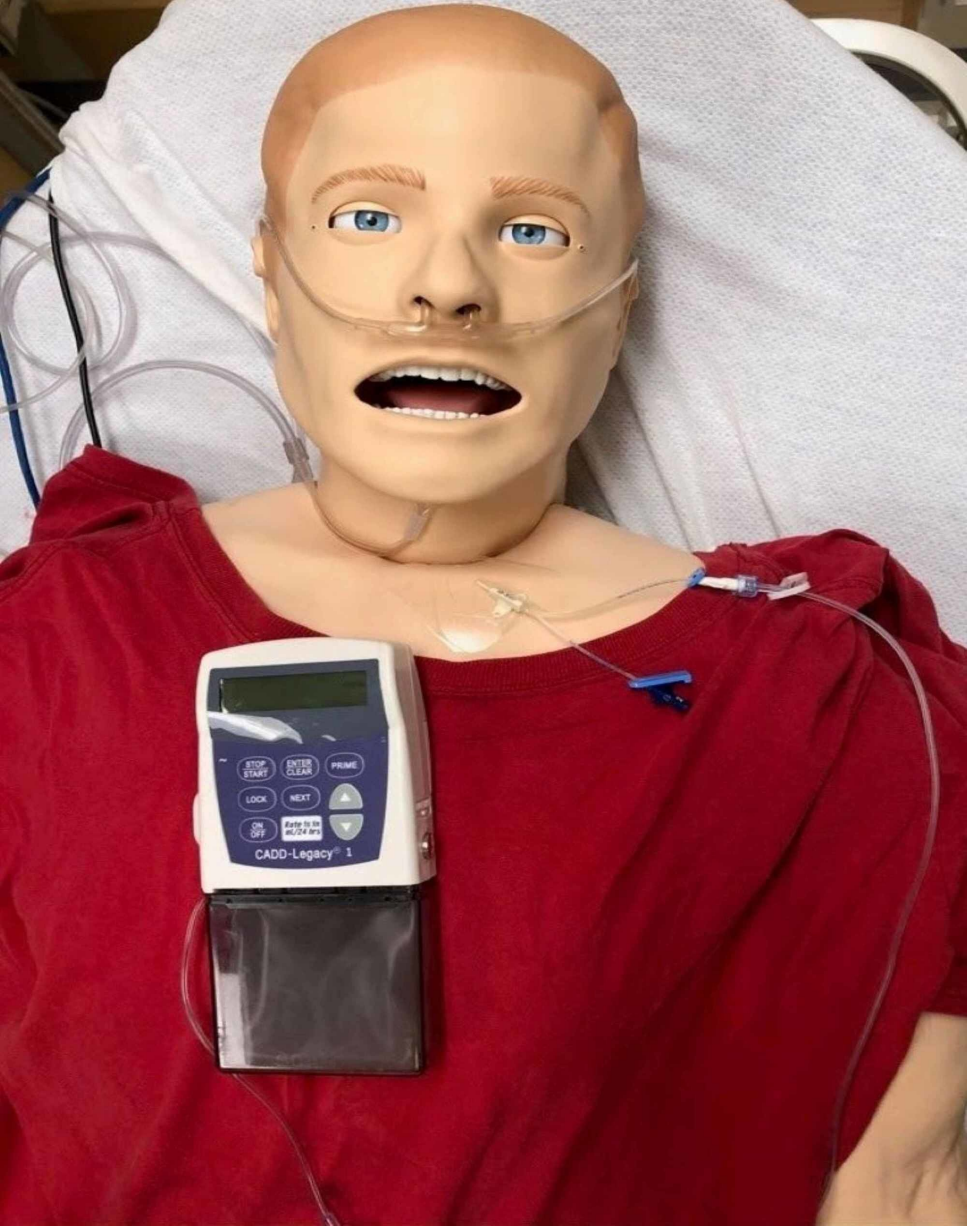
Intravenous Remodulin® pump mannequin setup Simulation mannequin setup with CADD-Legacy® 1 pump, IV tubing, central line supplies, and Tegaderm™ film

**Figure 3:**
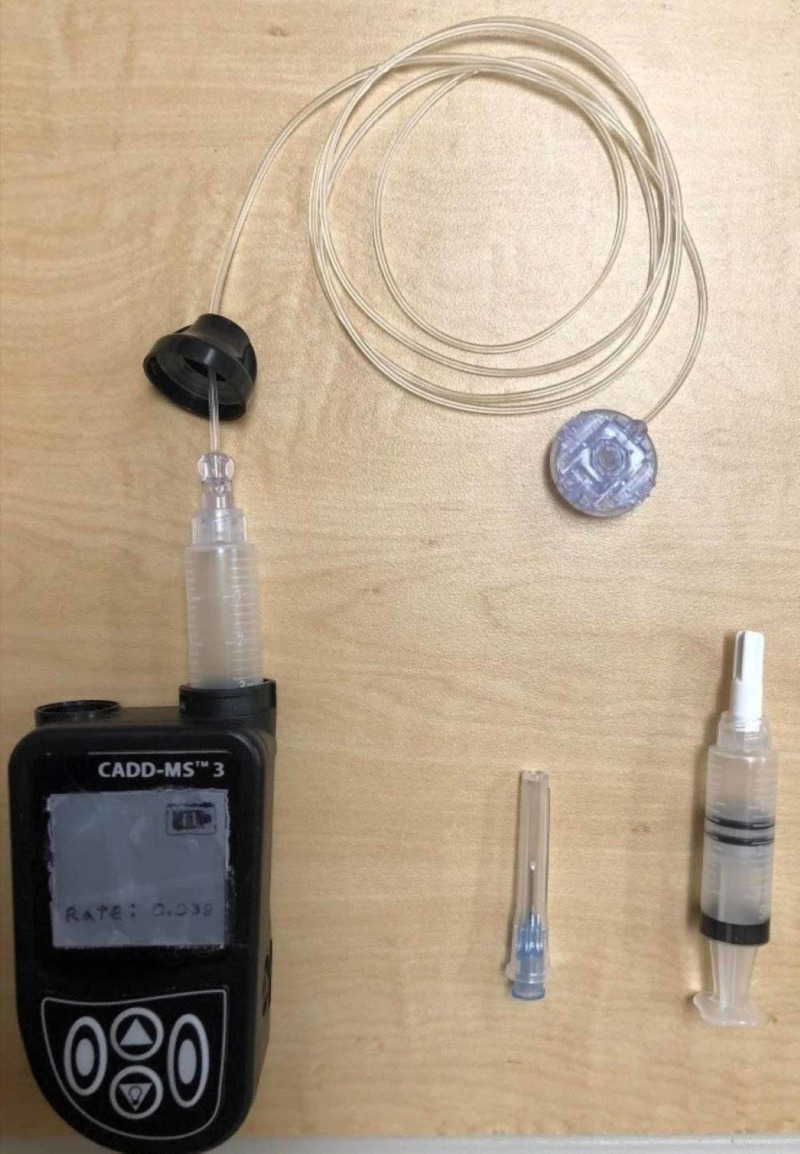
Subcutaneous Remodulin® pump supplies CADD-MS™ 3 pump with subcutaneous tubing and 3-mL medication syringe

**Figure 4:**
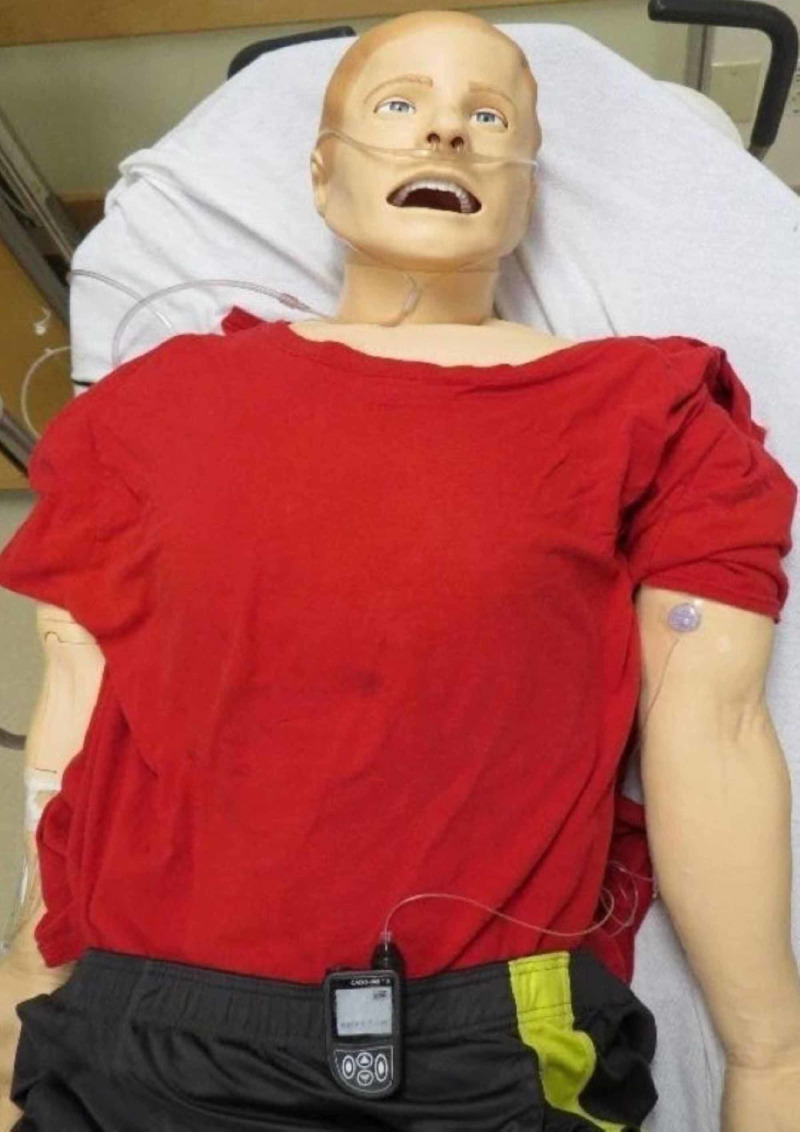
Subcutaneous Remodulin® pump mannequin setup Simulation mannequin with CADD-MS™ 3 pump setup, subcutaneous tubing, and 3-mL medication syringe

Along the patient’s left waistband, we secured the subcutaneous or IV Remodulin® pump, and connected this to the mannequin by the pump tubing. We obtained the CADD-Legacy® 1 (Smiths Medical, Inc., St. Paul, MN, USA) IV Remodulin® pump and the CADD-MS™ 3 (Smiths Medical, Inc., St. Paul, MN, USA) subcutaneous pump from our hospital’s PH team (Figures 1-4). The PH team has extra pumps available to coach new PH patients on how to replace medication cartridges and connect IV tubing to their access site.

Other options for potentially obtaining a pump for the simulation include contacting the manufacturer of Remodulin® directly or the pharmacies that supply Remodulin®. To maintain the appearance of a fully functional Remodulin® pump, we cut a 1 by 1 inch square piece of silver cardstock to fit to size and glued it onto the screen. On this cardstock, we drew the appearance of a full battery and the patient's continuous rate (Figure 3). We then covered the cardstock with a piece of clear plastic template (used in quilting supplies) and glued this directly above the cardstock. On the back of the pumps we taped the name of a pharmacy and phone number (our simulation center phone number), which, if noted, would allow the learner to call for the patient’s weight for dosing and concentration of medications. Most patients have this information on the back of their pumps as well.

Preparing the mannequin for the simulation

Materials Included

High-fidelity adult mannequin

Remodulin® pump - option A or option B

1 x 1 inch piece of silver cardstock

1 x 1 inch piece of clear plastic template

CADD® Extension Set - male luer, clamp, 0.2µ air-eliminating filter, and anti-siphon valve with male luer

3-mL Smiths Medical (St. Paul, MN, USA) medication syringe

Central IV line supplies

Tegaderm™ film (3M, Inc., St. Paul, MN, USA)

Application of IV Remodulin® CADD-Legacy® 1 pump to simulator - option A

Remove the white cap off the CADD-Legacy™ 1 IV Remodulin® pump

Remove the clear cap from the male end of the extension tubing and connect this to the female end of the CADD-Legacy™ 1 IV Remodulin® pump

Cut the central IV line near the white suture hub and cover it with Tegaderm™ onto the mannequin (Figures 1, 2)

Remove the blue cap from the other male end of the extension tubing and connect this to the female end of a central IV catheter

Fasten the Remodulin® pump to mannequin’s waistband

Application of subcutaneous CADD-MS™ 3 pump to simulator - option B

Remove the black cap off the CADD-MS™ 3 device

Remove the syringe tail from the 3-mL medication container

Place the 3-ml syringe body into the device with the male end up (Figure 3)

Attach the black cap over the 3-mL syringe

Attach the female end of the extension tubing onto the exposed male portion of the 3-mL syringe

Connect the subcutaneous portion of the tubing to the mannequin and cover it with Tegaderm™ (Figure 4)

Fasten the Remodulin® pump to mannequin’s waistband

Preparing the simulation scenario

**Figure 5:**
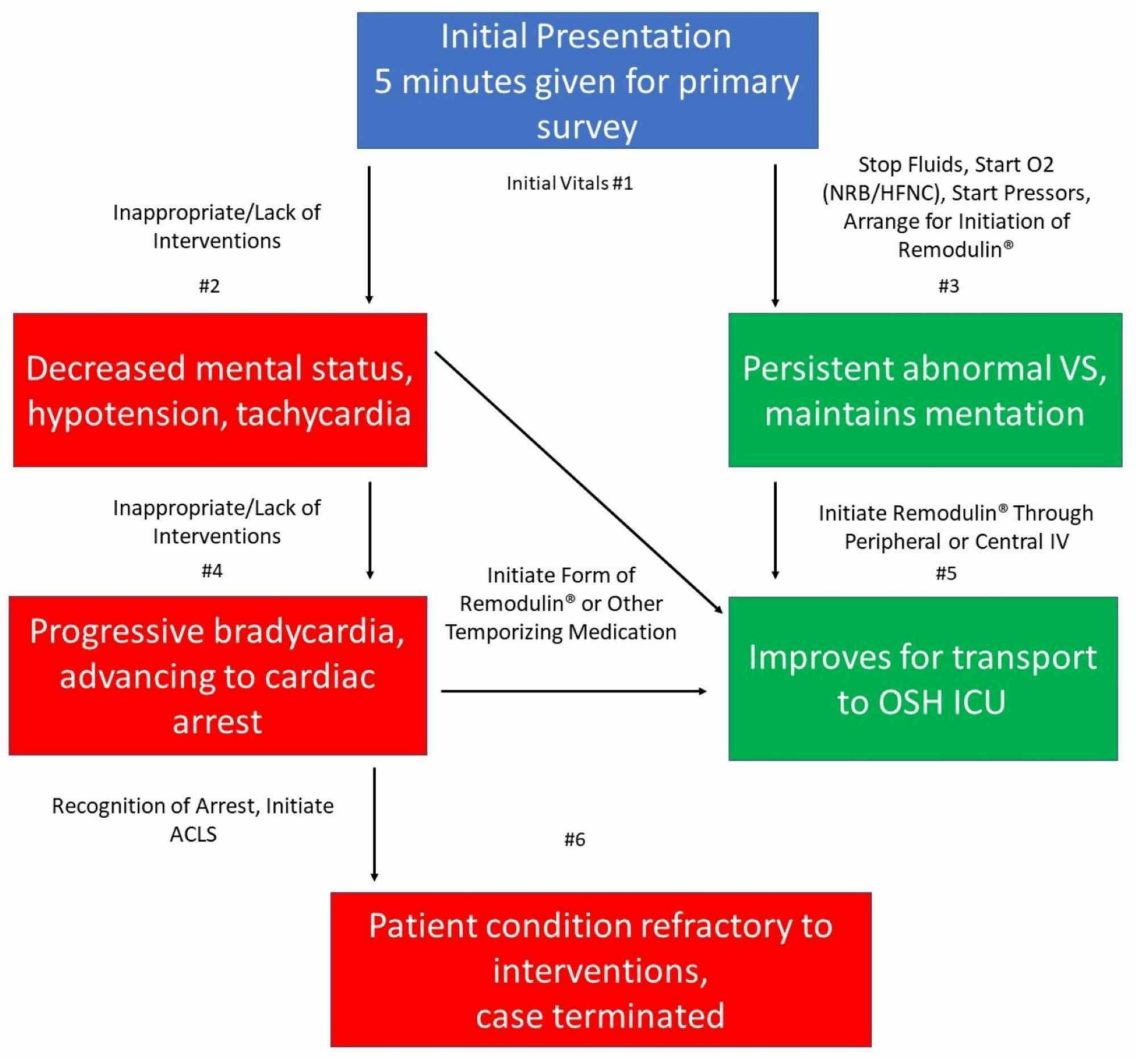
Flow chart showing correct and incorrect scenario branch points ACLS, advanced cardiac life support; NRB, non-rebreather; HFNC, high-flow nasal cannula; OSH, outside hospital; ICU, intensive care unit

**Figure 6:**
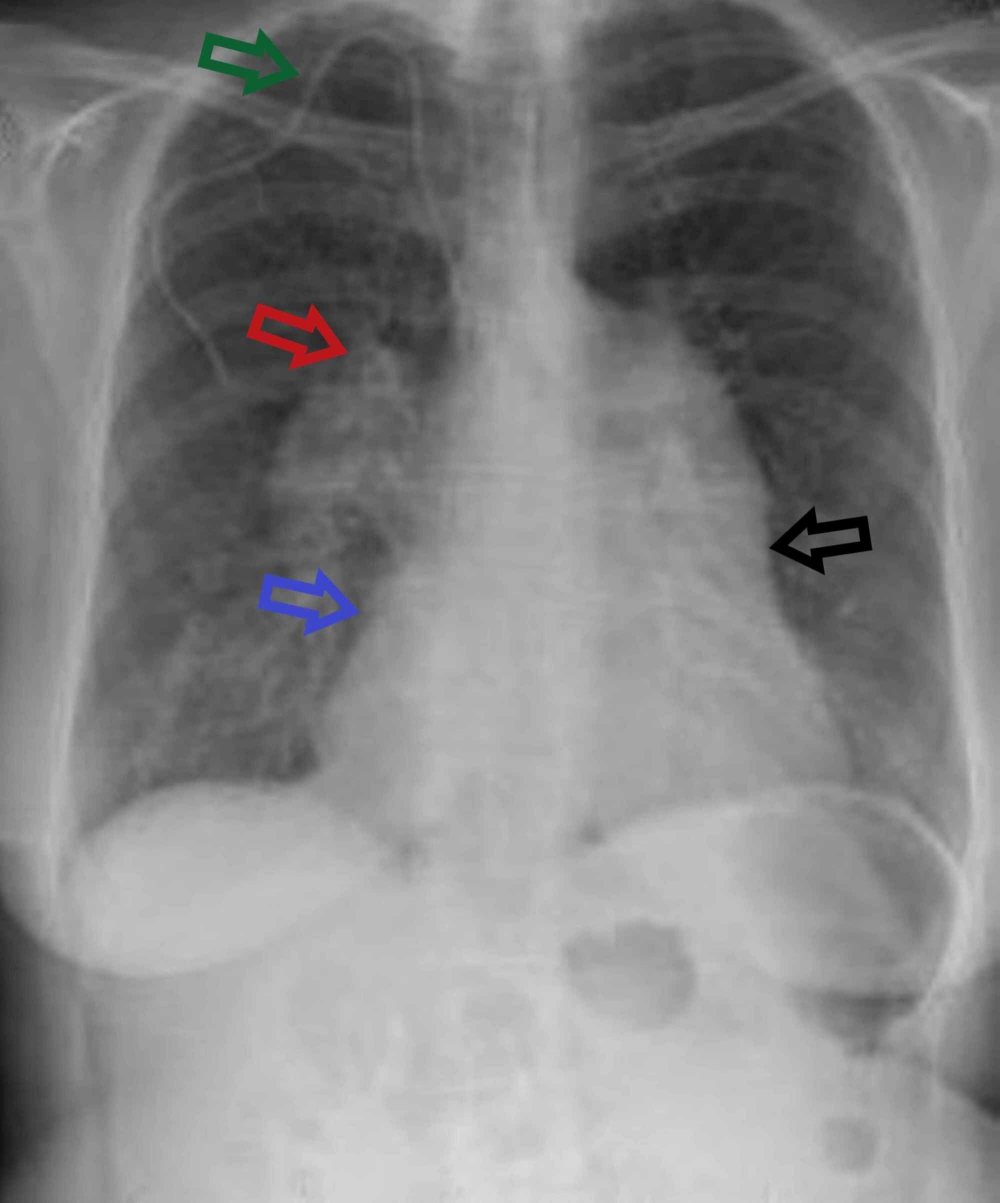
Chest X-ray of a patient with pulmonary hypertension The X-ray depicts cardiomegaly (black arrow), increased right ventricular diameter (blue arrow), pulmonary congestion (red arrow), and central venous access (green arrow)

**Figure 7:**
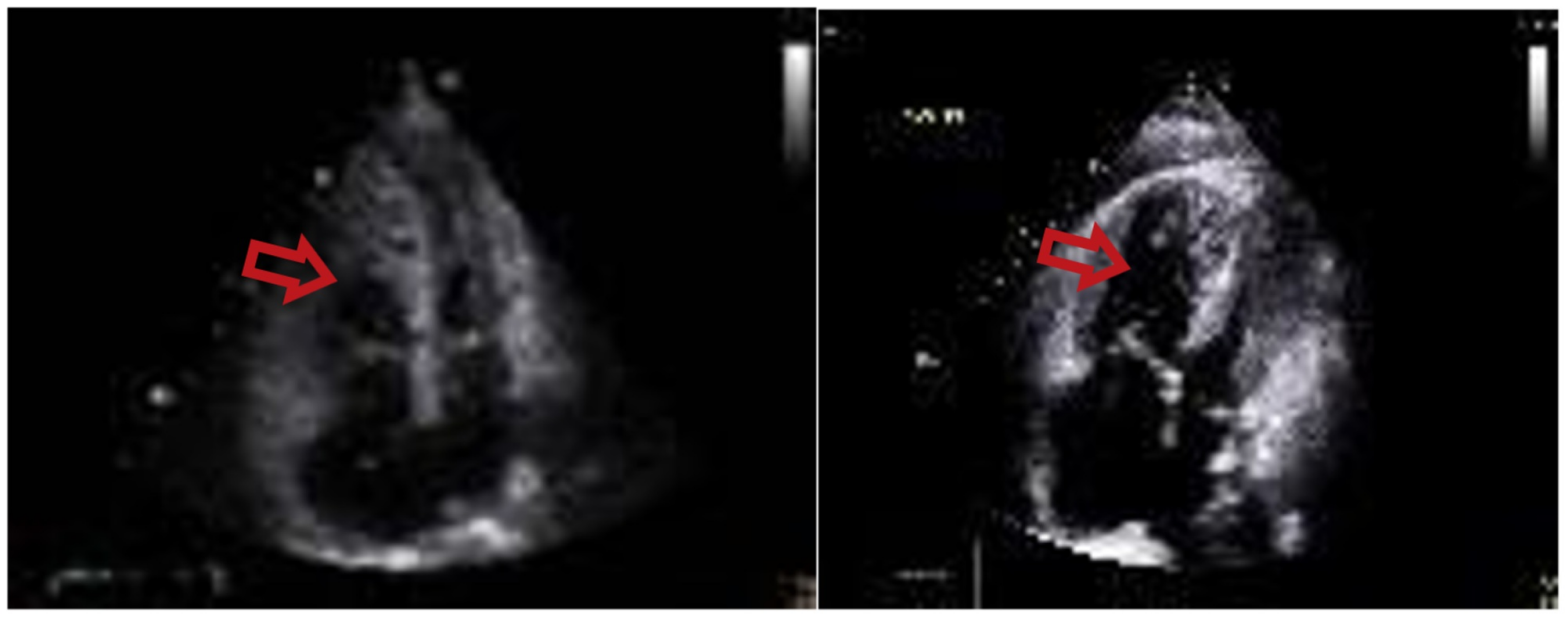
Echocardiogram of a patient with pulmonary hypertension The echocardiogram shows right ventricular dilation (red arrows)

**Figure 8:**
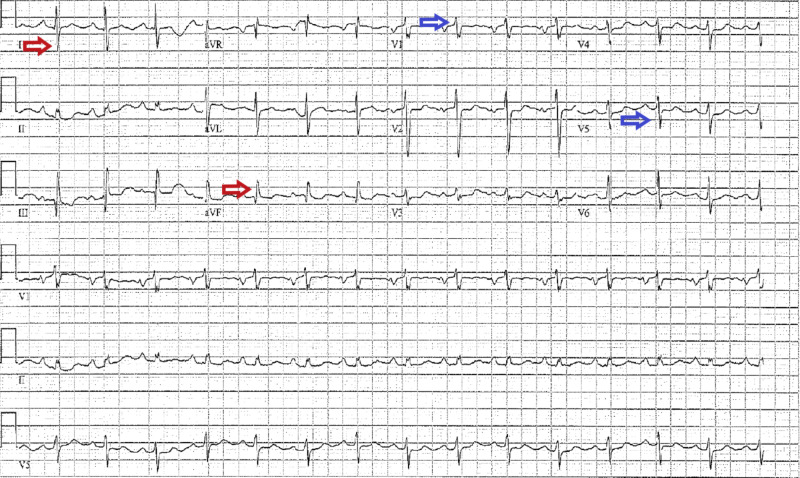
Electrocardiogram of a patient with pulmonary hypertension The electrocardiogram shows right axis deviation (red arrows), prominent R wave in V1 and S wave in V5 (blue arrows)

This case requires two or three embedded participants: a nurse and an emergency medical technician (EMT), with or without the addition of a family member to assist with history. A simulation technician is needed to run the case and voice the mannequin, and a faculty member should be present to lead the debriefing. All personnel should be pre-briefed and familiar with the case outline, the case flowchart, shown in Figure 5, the case branch point vital signs given in Table 1, and its associated clinical images (Figures 6-8). This case has several critical actions to stabilize the patient as demonstrated by the green pathway in the flowchart. The red pathway indicates improper interventions from which the learner has an opportunity to recover before the case is terminated.

**Table 1:** Vital signs NC, nasal cannula; HFNC, high-flow nasal cannula; NRB, non-rebreather; RR, respiratory rate

	Heart rate	Blood pressure	Temperature (°F)	O_2_ saturation	RR
#1	98	92/54	97.3	84% (4 L NC)	30
#2	106	80/48	97.3	80% (4 L NC)	34
#3	90	98/60	97.3	92% (>6 L HFNC or NRB)	25
#4	60	64/32	97.3	72% (4 L NC)	46
#5	80	104/68	97.3	96% (>6 L HFNC or NRB)	18
#6	0	0	97.3	Undetectable	0

Pre-briefing

Prior to initiating the case, all learners were pre-briefed on the mannequin’s capabilities and the resources available to them during the case. They were instructed to assign a team leader and to approach the mannequin as if this were a real patient. Given the complexity of the case, a broad differential must be worked through quickly and efficiently. We opted to deliver this simulation education during our upper level resident simulation curriculum.

Pulmonary hypertension case

A 37-year-old female with a history of mixed connective tissue disorder and stage IV PH presents by EMS with chief complaint of shortness of breath and chest pain. EMS reports that the patient was visiting her sister from out of town and has been in her usual state of health up until that morning when she began feeling short of breath and developed mid-sternal chest pain. When she stood up this morning, she felt light-headed and abruptly sat back down and called 911. Upon EMS arrival, the patient was alert and oriented but appeared in acute distress. Her vital signs initially revealed a blood pressure of 96/54 mm Hg, a heart rate of 95 bpm, a respiratory rate of 28, and an oxygen saturation of 79% on room air. She had coarse breath sounds throughout, had palpable pulses in all four extremities, and showed no focal neurologic deficits on their exam. EMS immediately obtained IV access in the patient’s right antecubital fossa and started a 1-L bolus of normal saline. They placed her on a 4-L nasal cannula with improvement of her oxygen saturation to 84%. EMS was able to obtain history from the patient and her sister who was on the way to the emergency department by private vehicle. Per EMS, the patient normally reports feeling a slight headache, body pains and an intense feeling of flushing since starting Remodulin®. Today, she noted that she has not had those symptoms. She denied headache, neck or back pain, radiation of her chest pain, fever, chills, nausea, vomiting, or abdominal pain.

Upon arrival to the emergency department, the patient continues to appear in respiratory distress and complains of mid-sternal chest pressure. During the history and physical, the patient is more lethargic and reports that it is hard for her to breathe. The patient is unable to provide further details due to increased work of breathing and lethargy. The patient’s medical history is otherwise negative, and EMS was unable to obtain surgical history. The patient did come with a medication list that includes Remodulin®, Adempas®, Opsumit®, and prednisone daily. There is no history of illicit drug usage and no known medical allergies. 

Optimal management of this case begins with the learner obtaining a focused history from the EMS team and patient. Pertinent positive details include the patient’s progressive shortness of breath, chest pain, and feeling of pre-syncope prior to arrival. Pertinent negative details include the sudden lack of sensation of her typical headache, body aches, and sensation of feeling flushed. The learner should then appropriately execute a targeted physical exam starting with the ABCs. Given the unique pathophysiology of PH patients, the learner will need to pay careful attention to correct resuscitation efforts that improve abnormal vital signs and physical exam findings. This includes stopping the IV fluids and ensuring appropriate, non-invasive means for oxygenating the patient. They should keep a broad differential and order other diagnostic studies like an electrocardiogram and chest X-ray. Learners should remain focused on the resuscitation of the patient throughout the case and not be distracted by the Remodulin® pump. Once initial stabilization efforts are achieved, the learner will need to promptly identify the cause of the patient’s symptoms and abnormal vital signs by recognizing an error in the Remodulin® delivery system. Early discussion with the hospital pharmacist or PH team should be done to arrange for the patient’s medication (or similar medication) to be restarted via peripheral or central IV access. Ultimately, IV access and the decision to provide appropriate medication should occur within the initial 5 to 10 minutes of patient presentation.

If the patient is taken to the CT scanner at any time (with persistently abnormal vital signs), rapid sequence intubation is performed, or the learner fails to recognize the need to restart the medication from a different IV site, the patient undergoes cardiac arrest and the case is terminated after a few additional minutes per the discretion of the teaching faculty member. We defer to the individual clinical experts when there are cases in which some missteps in management occur but the IV Remodulin is restarted. However, given the complexity of the case, we would lean towards successful management in most of those scenarios.

Debriefing

To complete the learning exercise, a debriefing session for all simulation participants was hosted immediately after termination of the case. Learners were challenged to review their own performance through an advocacy-inquiry debriefing approach. The team was also asked to reflect on their interprofessional communication and their clinical approach to an undifferentiated, unstable patient.

The main discussion focused on the management of the crashing PH patient with abnormal vital signs. The generation of a broad differential to guide workup and treatment served as the initial focus of the discussion as many learners were unfamiliar with this unique patient population. Deciphering normal from abnormal vital signs in PH patients was an area of difficulty for many learners due to limited knowledge of the baseline physiology for this patient population. Furthermore, resuscitation priorities are very unique for PH patients given complex hemodynamic balances. For example, in PAH and right ventricular dysfunction patients, hypotension typically represents hypervolemia, unless given a strong history of dehydration.

The discussion then switched to the Remodulin® pump itself and troubleshooting a faulty machine and/or line. In our experience, a vast majority of the learners had never seen a Remodulin® pump, let alone knew how to ensure proper functioning of an existing line. Additionally, learners were unfamiliar with the types of medication classes these patients may be taking, so a brief review of these concluded the debriefing.

Post-scenario didactics

After debriefing, the Remodulin® pump was circulated for residents to review and ask questions about aspects of the medication delivery and pump functions. Discussion reinforced key aspects and skills learned during the simulation. Emphasis was made around goals of resuscitation including limiting the amount of IV fluids, maximizing non-invasive routes of oxygenation, and identifying and treating sources of hypotension. Time was allotted at the end for learners to ask further questions. Two weeks after the case was presented, a grand rounds lecture was given by a member of the critical care faculty to reinforce knowledge and answer further questions about PH patient resuscitation.

## Discussion

PH is becoming an increasingly more common disease encountered by clinicians throughout various practice environments. With the rising age and growing population in the United States, it is expected that the number of PH patients will continue to grow. Clinicians in the emergency settings must cultivate a solid knowledge base in the subtleties of PH resuscitation, especially as advancements in acute therapies continue to increase. In our experience, many emergency medicine residents lack a firm grasp of the intricacies that must be known when caring for PH patients. Furthermore, there is a paucity of published information and didactics on caring for the acute complications that PH patients face. Due to the dire consequences of even slight missteps in treatment, simulation surrounding these topics will be essential for the training of our current and future residents.

Our simulation is structured to represent a high-acuity situation involving the quick decompensation of a hypotensive and tachycardic PH patient. We chose this case of medication and pump failure because it highlights the four most important aspects of caring for PH patients: fluid management and support of mean arterial blood pressure; airway management through maximization of non-invasive modes of ventilation; management of vasoactive medications (Remodulin®) and their abrupt cessation in the acute setting; and creation of a broad differential diagnosis including sepsis, heart failure, coronary artery disease, and other etiologies of a shock state. Through this simulation, we found that learners were generally unfamiliar with the Remodulin® pump and benefited from simulation activities surrounding its use.

## Conclusions

This case highlights the importance of education on the management of PH patients who present to the acute care setting in extremis. Learners were trained how to construct and work through a broad differential diagnosis and evaluate medication and pump failure. They developed expertise in focusing resuscitation efforts to limit fluids, initiate vasopressors early, emphasize non-invasive modes of ventilation, and avoid acidosis at all costs. Through high-fidelity simulation education, learners gained vital skills to incorporate into their clinical practice.

## References

[1] Hoeper MM, Bogaard HJ, Condliffe R (2013). Definitions and diagnosis of pulmonary hypertension. J Am Coll Cardiol.

[2] Foshat M, Boroumand N (2017). The evolving classification of pulmonary hypertension. Arch Pathol Lab Med.

[3] Hoeper MM, Humbert M, Souza R (2016). A global view of pulmonary hypertension. Lancet Respir Med.

[4] Shang X, Xiao S, Dong N (2017). Assessing right ventricular function in pulmonary hypertension patients and the correlation with the New York Heart Association (NYHA) classification. Oncotarget.

[5] (2020). Learn about pulmonary arterial hypertension. Scientific and Medical.

[6] Rubin LJ (2004). Diagnosis and management of pulmonary arterial hypertension: ACCP evidence-based clinical practice guidelines. Chest.

[7] Kumar P, Thudium E, Laliberte K, Zaccardelli D, Nelsen A (2016). A comprehensive review of treprostinil pharmacokinetics via four routes of administration. Clin Pharmacokinet.

[8] Simonneau G, Barst RJ, Galie N (2002). Continuous subcutaneous infusion of treprostinil, a prostacyclin analogue, in patients with pulmonary arterial hypertension. Am J Respir Crit Care Med.

[9] Wade M, Baker FJ, Roscigno R, DellaMaestra W, Arneson CP, Hunt TL, Lai AA (2004). Pharmacokinetics of treprostinil sodium administered by 28-day chronic continuous subcutaneous infusion. J Clin Pharmacol.

[10] Implantable System for Remodulin® (2017 (2019). Implantable system for Remodulin®. https://www.accessdata.fda.gov/cdrh_docs/pdf14/P140032C.pdf.

[11] (2019). Remodulin® (treprostinil): pulmonary arterial hypertension (PAH) treatment. Prescribing information. http://www.remodulin.com/downloads/remodulin-prescribinginformation.pdf.

[12] Kingman MS, Tankersley MA, Lombardi S (2010). Prostacyclin administration errors in pulmonary arterial hypertension patients admitted to hospitals in the United States: a national survey. J Heart Lung Transplant.

[13] Stamm JA, Risbano MG, Mathier MA (2011). Overview of current therapeutic approaches for pulmonary hypertension. Pulm Circ.

